# Time-sequential fibroblast-to-myofibroblast transition in elastin-variable 3D hydrogel environments by collagen networks

**DOI:** 10.1186/s40824-023-00439-x

**Published:** 2023-10-17

**Authors:** Nhuan T. Do, Sun Young Lee, Yoon Seo Lee, ChaeHo Shin, Daeho Kim, Tae Geol Lee, Jin Gyeong Son, Se-Hwa Kim

**Affiliations:** 1 Safety Measurement Institute, Korea Research Institute of Standards and Science, 267 Gajeong-Ro, Yuseong-Gu, Daejeon, 34113 Republic of Korea; 2 BioMedical Measurement, University of Science and Technology, 217 Gajeong-Ro, Yuseong-Gu, Daejeon, 34113 Republic of Korea; 3 Interdisciplinary Materials Measurement Institute, Korea Research Institute of Standards and Science, 267 Gajeong-Ro, Yuseong-Gu, Daejeon, 34113 Republic of Korea; 4 Nanoconvergence Measurement, University of Science and Technology, 217 Gajeong-Ro, Yuseong-Gu, Daejeon, 34113 Republic of Korea; 5 Bruker Nano Surface & Metrology, Bruker Korea, Seongnam, 13493 Republic of Korea

**Keywords:** Elastin, Collagen, Fibroblast-to-myofibroblast transition, Calcium signaling

## Abstract

**Background:**

Fibrosis plays an important role in both normal physiological and pathological phenomena as fibroblasts differentiate to myofibroblasts. The activation of fibroblasts is determined through interactions with the surrounding extracellular matrix (ECM). However, how this fibroblast-to-myofibroblast transition (FMT) is regulated and affected by elastin concentration in a three-dimensional (3D) microenvironment has not been investigated.

**Methods:**

We developed an insoluble elastin-gradient 3D hydrogel system for long-lasting cell culture and studied the molecular mechanisms of the FMT in embedded cells by nanoflow LC–MS/MS analysis along with validation through real-time PCR and immunofluorescence staining.

**Results:**

By optimizing pH and temperature, four 3D hydrogels containing fibroblasts were successfully fabricated having elastin concentrations of 0, 20, 50, and 80% in collagen. At the low elastin level (20%), fibroblast proliferation was significantly increased compared to others, and in particular, the FMT was clearly observed in this condition. Moreover, through mass spectrometry of the hydrogel environment, it was confirmed that differentiation proceeded in two stages. In the early stage, calcium-dependent proteins including calmodulin and S100A4 were highly associated. On the other hand, in the late stage after several passages of cells, distinct markers of myofibroblasts were presented such as morphological changes, increased production of ECM, and increased α-SMA expression. We also demonstrated that the low level of elastin concentration induced some cancer-associated fibroblast (CAF) markers, including PDGFR-β, and fibrosis-related disease markers, including THY-1.

**Conclusion:**

Using our developed 3D elastin-gradient hydrogel system, we evaluated the effect of different elastin concentrations on the FMT. The FMT was induced even at a low concentration of elastin with increasing CAF level via calcium signaling. With this system, we were able to analyze varying protein expressions in the overall FMT process over several cellular passages. Our results suggest that the elastin-gradient system employing nonlinear optics imaging provides a good platform to study activated fibroblasts interacting with the microenvironment, where the ECM plays a pivotal role.

**Graphical Abstract:**

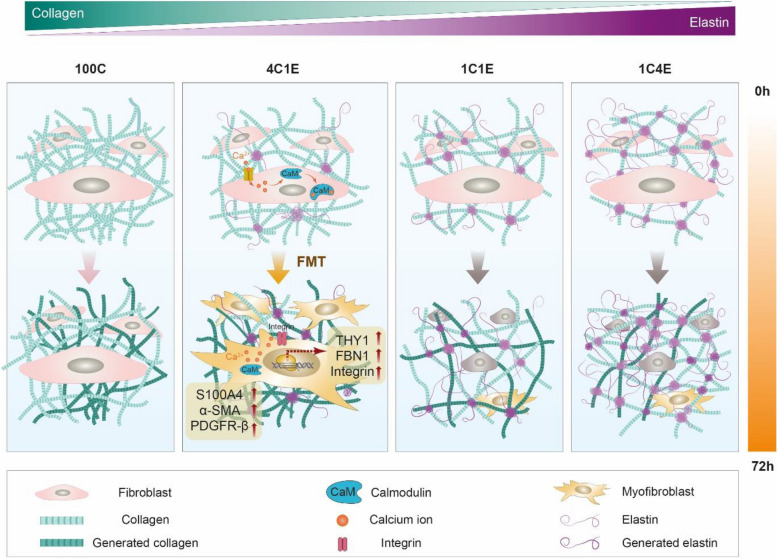

**Supplementary Information:**

The online version contains supplementary material available at 10.1186/s40824-023-00439-x.

## Introduction

Fibrosis, governed by fibroblasts, plays a crucial role in both normal physiological and pathological phenomena [1]. Fibroblasts residing in healthy tissues in a quiescent status reciprocally respond to physical demands by synthesizing, degrading, and remodeling the surrounding extracellular matrix (ECM). By controlling the ECM in connective tissues, fibroblasts contribute to maintaining tissue homeostasis in response to positive and negative feedbacks. Dormant fibroblasts can be activated to facilitate repair and regeneration during wound healing, tissue inflammation, and pathological conditions [2]. This process corresponds to the fibroblast-to-myofibroblast transition (FMT) in wound healing and several types of inflammation. Broadly, the stages of the FMT can be sub-divided by the cellular type: proto-myofibroblasts and myofibroblasts. Proto-myofibroblasts, in the initial transition state, are known to characterize the rearrangement of the actin cytoskeleton and the formation of focal adhesions [3]. The in vivo activating factors for proto-myofibroblasts are not well understood because one crucial factor in inducing proto-myofibroblast formation is mechanical tension [4]; thus, proto-myofibroblasts have mainly been studied in in vitro environments that facilitate mechanical tension via attachment to the plastic bottom of tissue culture dishes. Proto-myofibroblasts can develop into differentiated myofibroblasts via the following local events: exposure to TGF-β1 and changes in the mechanical properties of the ECM. The absence of α-smooth muscle actin (α-SMA) in proto-myofibroblasts is the distinct difference from myofibroblasts [3–5]. Myofibroblasts and cancer-associated fibroblasts (CAFs) share similar common characteristics such as stellate phenotype, the expression of α-SMA, and the production of collagen [6]. Another important common feature is that they are highly associated and controlled by the surrounding microenvironment by secreting ECM proteins such as collagen, laminin, elastin, and fibronectin. These ECM components provide structural support as well as mediate cellular signaling and interactions. Therefore, extensive investigations into differentiated fibroblasts including myofibroblasts and CAFs associated with the ECM microenvironment have been carried out for the purpose of potential therapeutic strategies.

Recently, the application of hydrogels as substitutes for the ECM has garnered substantial attention due to their physical characteristics that closely mirror the native ECM structure, as demonstrated in both in vitro and in vivo studies. Hydrogels are typically categorized into two classes based on their origin: natural and synthetic. Synthetic polymers commonly include substances such as poly(ethylene glycol), while natural polymers frequently involve collagen and elastin. Owing to their inherent biocompatibility, which promotes cell growth and proliferation, natural hydrogels are predominantly utilized in medical applications [7, 8]. Various methods including freeze-drying, electrospinning, micropatterning, and bulk polymerization have been utilized in the fabrication of ECM mimics, where the method of choice depends on the intended application. For instance, in the fabrication of artificial muscles and valves, freeze-drying and electrospinning methods are typically preferred due to their ability to create two-dimensional (2D) ECM mimic scaffolds [7]. These methods involve limitations, though, in the application of 2D ECM mimic scaffolds in various biomedical fields due to their inability to accommodate embedded cells during the fabrication process, which often involves exposure to extremely low temperatures or high voltages at room temperature for extended periods [9]. To overcome these limitations, bioprinting has been suggested for the fabrication of a three-dimensional (3D) ECM mimic, or hydrogel, which is capable of supporting cellular growth during the fabrication process. But hydrogels fabricated by bioprinting still face the challenge of maintaining cell viability during fabrication, despite their ability to closely mimic the natural ECM conditions [10]. Among these methods mentioned above, bulk polymerization has the potential for the development of a 3D cell-friendly environment. Although this method requires high concentrations of monomers and cross-linkers, polymerization by only physicochemical factors (e.g., temperature and pH) is the most biocompatible and adjustable for long-lasting cell culture experiments [7, 10, 11]. Difficulties remain, however, in the characterization of hydrogels fabricated by bulk polymerization due to their opacity, which can be improved by applying nonlinear optics imaging [12].

Collagen as a fibril protein is the most abundant protein in the ECM providing tissue strength and stiffness [13]. Due to its high biocompatibility and abundance in the body, collagen has been extensively utilized in tissue engineering. To develop more improved materials with a high similarity to native tissue, the inclusion of other ECM components, including hyaluronic acid [14, 15] and elastin [13, 16, 17], to collagen-based constructs has been investigated. While collagen mainly determines the mechanical properties of tissue, elastin in particular provides elasticity and recoil of tissues from stretching. Besides this role in controlling the mechanical properties of tissue, elastin is involved in aging and pathological process as well [18]. This is due to the features of elastin, namely a high hydrophobicity and cross-linked structure, which lead to elastin exhibiting no turnover in healthy tissue with a long half-life of over 70 years. Therefore, elastin is capable of cellular regulatory functions and plays an important role in microenvironment control. In a normal physiological state, elastin concentration is generally lower than 10% except in specific organs such as blood vessels [19]. Substantial research has been conducted on what concentrations of elastin cause pathological states. Recently, at 10% elastin in a collagen-based ECM analogue, the FMT of laden cells was induced by exerting mechanical loadings [20]. Different cell types other than fibroblasts, such as smooth muscle cells, were also differentiated in elastin-containing 2D collagen gels [21]. In an in vivo study, the elastin content in ECM was increased in the case of intervertebral disc degeneration, a condition characterized by chronic inflammation often leading to tissue fibrosis [22, 23]. Although these previous reports suggested that the elastin content is quite important to control the axis of physiological and pathological conditions, the question of how much elastin content induces pathological responses with underlying molecular mechanisms still remains unanswered.

In the present study, we hypothesized that different elastin concentrations would induce different cellular responses in a 3D environment. The aim of this study was thus to elucidate the influence of elastin concentration on fibroblast differentiation and to examine the molecular mechanisms involved. We prepared a long-lasting cell culture 3D collagen hydrogel system with an elastin gradient, which is composed of hydrogels with different ratios of collagen and elastin: 100% collagen (100C), 80% collagen: 20% elastin (4C1E), 50% collagen: 50% elastin (1C1E), and 20% collagen: 80% elastin (1C4E). Due to the opacity of thick bulk polymerized hydrogels, we utilized nonlinear optics imaging technologies for the characterization of the ECM structures and cellular behavior inside the hydrogels, which could image collagen and elastin in a noninvasive manner. Molecular mechanisms were investigated via mass spectrometry in time course. Then we assessed the effect of 20% elastin on the FMT by validating gene and protein expression via cell staining, and also measured the modulus of all hydrogels via atomic force microscopy. This study suggests that the elastin-induced FMT needs to be considered in 3D conditions with long-term cultivation (over 3–4 passages) owing to time-sequential cellular proliferation changes.

## Materials and methods

### Materials

Tris–HCl buffer (pH 8.0), phosphate buffered saline (PBS), sodium chloride (NaCl), formic acid (FA), ammonium bicarbonate (AmBic), dithiothreitol (DTT), iodoacetamide (IAA), L-cysteine, acetic acid (Sigma, #45754-100ml), sodium bicarbonate (Sigma, #144-55-8), 4% formaldehyde (Sigma, #HT5011), triton X-100 (Sigma, #X100), normal horse serum (Sigma, #H1138), DAPI (Sigma, #9542), and collagenase (Sigma, #C0130) were purchased from Sigma-Aldrich (St. Louis, MO, USA). Protease inhibitor cocktail was purchased from Roche Diagnostic GmbH (Mannheim, Germany). The HLB cartridges were purchased from Waters (MA, USA). Acetonitrile (ACN) (with 0.1% FA), water (with 0.1% FA), n-dodecyl beta-D-maltoside (DDM), trypsin, and bicinchoninic acid (BCA) protein assay reagent were purchased from Thermo Fisher Scientific (Rockford, IL, USA). Rat tail collagen type I solution (Biomatrix, #5010) was purchased from Advanced Biomatrix (Carlsbad, CA, USA). BrdU primary antibody (Cell signaling, #5292) was purchased from Cell Signaling Technology (MA, USA), and mouse anti-S100A4 primary antibody was purchased from Invitrogen (Invitrogen, #MA5-31332). Anti-β-tubulin primary antibody (Abcam, #ab15568), anti-PDGFR-β primary antibody (Abcam, #ab32570), and anti-α-SMA primary antibody (Abcam, #7817) were purchased from Abcam (Cambridge, UK). Alexa Fluor 549 (Thermo Fisher #A21203, #A21207) and Alexa Fluor 488 (Thermo Fisher, #A11029) were purchased from Thermo Fisher Scientific (MA, USA). A Sircol™ inSoluble Collagen Assay kit (Biocolor, #S6000) and Fastin – Elastin assay kit (Biocolor, #F4000) were purchased from Biocolor (Co Antrim, UK). Finally, recombinant human TGF-β protein (Biotechne, #240-B-010) was purchased from Bio-Techne (MN, USA).

### Fabrication of fibroblast-laden collagen hydrogels with four different gradients of elastin

Hydrogels were fabricated according to previously reported methods after cell-friendly optimization [21]. First, insoluble elastin from bovine neck ligament (Sigma, #1625) was suspended in 0.1 M acetic acid (Sigma, #45754 100 ml) at 25 mg/ml as the elastin stock solution (ES). Rat tail collagen type I solution (Biomatrix, #5010) at a concentration of 6.2 mg/ml was used as the collagen stock solution (CS). The four types of elastin-variable hydrogels (100C, 4C1E, 1C1E, 1C4E) were fabricated by mixing appropriate volumes of CS, ES, completed cell culture medium, and 7.5% sodium bicarbonate (Sigma, #144-55-8). The pH of the mixture was adjusted to 7.4, the final concentration of collagen was maintained at 2 mg/ml in each hydrogel, and the final concentration of elastin was varied (0–8 mg/ml) based on the collagen/elastin ratios of 1:0, 4:1, 1:1, and 1:4, respectively. Then the mixture was added into a 96-well plate at a volume of 35 µl/well and incubated in an incubator (37 °C, 5% CO_2_) for 2 h to allow gelation. The hydrogels, without cells, were then fixed with 4% formaldehyde (Sigma, #HT5011) for 30 min and washed with 1X PBS several times before storage at 4 °C until further analysis. After the hydrogel components were mixed with a neutralization solution, human dermal fibroblasts (HDFs) at passage 7 were added at a density of 1 × 10^5^ cells/ml. Then 35 μl of hydrogel mixture, including cells, was gently added into a 96-well plate to avoid bubbles. Finally, gelation of the hydrogel mixture was induced in an incubator (37 °C, 5% CO_2_) for 2 h. Completed cell culture media with or without TGF-β or BAPTA-AM was added to each well for a fibroblast culture within hydrogels.

### Morphological characterization by scanning electron microscopy imaging

The hydrogels were dehydrated by washing with ethanol and freeze-dried for 6 h (FD8512 Floor, ilShinBioBase, Rep. of Korea). Then the dried hydrogels were coated with an ultrathin layer of gold using an ion sputter coater (SPT-20, Coxem, Rep. of Korea) set at 4 mA for 120 s and visualized with a Hitachi S-4800 scanning electron microscope operating at 10 kV (FESEM, S-4800, Hitachi, Japan).

### Assessment of cell proliferation in 3D gels with 5-Bromo-2-deoxyuridine (BrdU) assay

To measure fibroblast proliferation, we used a BrdU assay as described in a previous report [24]. First, 1 × 10^5^ cells/ml of fibroblasts were seeded into hydrogel mixtures in a 96-well plate. After gelation, completed cell culture medium containing 10 μM BrdU was added into each well. Cells were continuously cultured up to 72 h. At a certain period of time, hydrogels with or without BrdU-treated cells were fixed using 4% formaldehyde (Sigma, #HT5011). After being washed with PBS 1X three times (30 min each time), the hydrogels were washed with 0.1% Triton X-100 (Sigma, #X100) and incubated for 30 min in 0.1% Triton for permeabilization. Then the cells inside the hydrogels were blocked with 5% normal horse serum (Sigma, #H1138) for 1 h. Hydrogels were incubated with mouse anti-BrdU primary antibody (1:200, Cell Signaling, #5292) overnight at 4 °C. Next, hydrogels were washed with 0.1% Triton X-100 three times (30 min each time). After washing, the hydrogels were incubated with Alexa Fluor 549 (1:200, Abcam #A21203) for 1 h. Then, the remaining dye in hydrogels was washed with 0.1% Triton X-100 and hydrogels were subsequently stained with DAPI (Sigma, #9542) for 15 min. Cell imaging was performed using a laser scanning confocal microscope (LSCM, Olympus FV3000) and Olympus FluoView FV10-ASW software. To observe the fluorescence signals from the cells, 5.0 μm thick images in a z-stack picture were analyzed. All samples were imaged using identical exposure times and laser power settings. Qualification imaging analysis was done using IMARIS 7.6 software.

### Microscopic characterization of collagen and elastin in elastin-variable hydrogels with nonlinear optics imaging

A previously developed method was used for imaging collagen fibers employing second harmonic generation (SHG) signals [25]. Collagen fibers in the hydrogels were observed via SHG signals via setting the wavelength of a fs pulsed laser to 810 nm. Briefly, HDFs cultured inside fixed hydrogels were permeabilized using 0.1% Triton X-100 and then blocked with 5% normal horse serum in 1X PBS containing 0.1% Triton X-100 for 90 min. Rabbit anti-β-tubulin primary antibody (1: 200, Abcam, #15568) was incubated overnight with the hydrogels at 4 °C. The hydrogels were then were washed 3 times (30 min each time) with 0.1% Triton X-100 in PBS 1X. After incubating the hydrogels with a secondary antibody conjugated with Alexa Fluor 594 (1:200, Thermo Fisher, #A21207) for 60 min at room temperature, the hydrogels were washed with PBS 1X twice (30 min each time) and the collagen fibers were observed using multimodal nonlinear optical (MNLO) microscopy and SHG signals setting the wavelength of the fs pulsed laser to 810 nm. Z-depth images were set at a thickness of 5.0 μm. All images were obtained using FluoView software (FV10-ASW; Olympus Corp., Tokyo, Japan).

### Measurement of collagen fiber length

Measurements of collagen fiber length were carried out following a previously reported method using IMARIS 7.4 software [26]. All SHG images were converted to black and white images and set at the same threshold level, which allowed for the separation of collagen fibers. After that, the freehand tool was utilized to measure the length of the collagen fiber. Approximately 25 fibers were randomly selected for each measurement.

### Measurement of collagen by colorimetric assay

Collagenase (Sigma, #C0130) was used to digest the hydrogels. After hydrogel gelation, 100 µl of 1 mg/ml collagenases was added to the hydrogels in culture medium followed by incubation at 37 °C until all hydrogels were digested. Then the collagen level was measured using the Sircol™ inSoluble Collagen Assay kit based on Sircol dye reagent, which binds the basic groups of soluble collagen molecules. Following manufacturer’s instructions, collagen in the digested hydrogel mixture was isolated using cold isolation and concentration reagent (200 μl/sample). After vortexing to mix the contents for a few minutes every 15 min, the mixture was left at 4 °C for 30 min. The mixture was centrifuged at 12,000 rpm for 10 min. The collagen pellet was retained and 1000 μl of cold diluted acid-salt wash reagent was added to each sample to wash the collagen pellet. The collagen after collection using a centrifuge (12,000 rpm, 10 min) was labeled by adding Sircol dye reagent (1000 μl each sample). Samples were gently shaken for 30 min, and the collagen pellet was collected after centrifugation (12,000 rpm, 10 min). Then any unbound dye from the surface of the collagen pellet was washed off by adding 750 μl of ice-cold acid-salt wash reagent into each sample. After centrifugation (12,000 rpm, 10 min), an alkali reagent was added to each sample to solubilize the collagen (250 μl). The absorbance was observed at 550 nm using a BioTek Synergy H1 microplate reader (BioTek Instruments, Inc., USA).

### Measurement of elastin by colorimetric assay

Hydrogels were digested using 1 mg/ml collagenase at 37 °C for around 1 h. Then an elastin measurement assay was performed following the manual of the manufacturer using a Fastin – Elastin assay kit (Biocolor, #F4000). Briefly, insoluble elastin in the mixture was extracted using 0.25 M oxalic acid while heating at 100 °C for 60 min. After cooling down, the mixture was centrifuged at 12,000 rpm for 10 min. The supernatant after centrifugation was saved and mixed with an equal volume of elastin-precipitating reagent. After 10 min, the mixture was centrifuged at 12,000 rpm for 10 min. The supernatant was carefully poured out and the pellet was retained. Dye reagent was added to the pellet at a volume of 1 ml per sample. Then the mixture was vortex mixed and incubated for 90 min at room temperature. After drying, the mixture was centrifuged at 12,000 rpm for 10 min to remove any remaining dye reagent. Finally, dye dissociation reagent was added into each tube. After mixing well, the mixture was transferred to a 96-well plate and read with a BioTek Synergy H1 Microplate Reader at a wavelength of 513 nm.

### Nanoflow LC–ESI–MS/MS analysis

To extract the protein from HDFs, HDF cell pellets of each condition were mixed with 100 μl of a 0.2% DDM solution containing 150 mM NaCl, 50 mM Tris–HCl, and one tablet of protease. The cell mixtures were mixed vigorously and dissolved at 95 °C for 10 min. Then the solution was centrifuged at 12,000 rpm for 15 min to remove cell debris and take the supernatants. The extracted proteins in the supernatant were subjected to a BCA assay to measure total protein concentration.

Twenty micrograms of protein were reduced using a 50 mM AmBic solution containing 10 mM DDT. To prevent re-folding of the disulfide bonds, 40 nM IAA was added and reacted at room temperature for 30 min. Thereafter, trypsin (protein/enzyme = 20:1) was added to the solution and digested at 37 °C for 18 h to form peptides. Utilizing an HLB cartridge for purification, the peptide solution was then dried under vacuum. The dried sample was stored at –20 °C before nanoflow liquid chromatography–electrospray ionization–tandem mass spectrometry (nLC-ESI–MS/MS).

A solution of H_2_O/ACN (98:2, v/v) containing 0.1% FA was used to reconstitute the dried samples, and 250 ng of the resulting peptide mixture was introduced into a NanoElute LC system connected to a hybrid trapped ion mobility spectrometry–quadrupole time-of-flight mass spectrometer (timsTOF Pro, Bruker Daltonics, Bremen, Germany) equipped with a modified nano-electrospray ion source (CaptiveSpray, Bruker Daltonics). In a handmade column (75 μm inner diameter, 250 mm length) packed with C18 resins (1.9 μm, 120 Å, Dr. Maisch, Germany), the peptide mixtures were separated at 50 °C with a constant flow of 400 nl/min and then eluted using the following binary gradient of mobile phases A (0.1% FA in H_2_O) and B (0.1% FA in ACN): 2% to 17% B for 45.0 min, 17% to 25% for 22.5 min, 25% to 37% for 7.5 min, 37% to 80% for 5.0 min, and then kept at this level for 10 min to rinse the analytical column. The timsTOF Pro device was operated in parallel accumulation serial fragmentation (PASEF) acquisition mode using Bruker Compass HyStar 5.0.37.1. The settings for MS and MS/MS scans were as follows: mass range of 100 to 1700 m/z, 1/K_0_ start at 0.6 Vs/cm^2^ and end at 1.6 Vs/cm^2^, capillary voltage of 1500 V, dry gas flow rate of 3 l/min, and dry temperature of 180 °C; PASEF mode: 10 MS/MS scans (total cycle time 1.16 s), charge range of 0 to 5, active exclusion for 0.4 min, scheduling target intensity of 20,000, and intensity threshold of 2500, depending on precursor mass and charge.

### Proteomic data analysis

The obtained raw data were submitted to PEAKS Studio 10.5 (Bioinformatics Solutions, Waterloo, Canada) for protein identification and label-free quantification (LFQ) searches against the SwissProt database of *Homo sapiens* (human, UP00000564, downloaded 22/11/2019, 20,379 entries) from Uniprot (www.uniprot.org/) with a false discovery rate (FDR) of 0.01. The search parameters for identification were as follows: (a) trypsin as the specific enzyme, two missed cleavages allowed; (b) fixed modification: carbamidomethylation of cysteine, and variable modification: oxidation of methionine and acetylation of protein N-term, allowing for three variable post-translational modifications per peptide; (c) precursor mass error tolerance of 20.0 ppm; (d) fragment mass error tolerance of 0.05 Da. Following the completion of protein identification, LFQ was performed using the analyzed PEAKS dataset. The analysis of variance (ANOVA) method was used to conduct the LFQ analysis, and the significance thresholds were set at two unique peptides, a data filter in at least two samples per group, a significance of 20 (*p* value = 0.01), and a 1.5-fold change. Total ion chromatography (TIC) was used to perform data normalization. GO term enrichment of HDFs was carried out using the ShinyGO program (version 0.77) after the data were exported to Microsoft Excel.

### Immunofluorescence-based cell staining

The proteins S100A4, PDGFR-β, and α-smooth muscle actin (α-SMA) were identified via immunofluorescence staining. After being washed and permeabilized with PBS 1X and 0.1% Triton X-100 respectively, the 100C and 4C1E hydrogels were incubated overnight with the following primary antibodies: mouse anti-S100A4 primary antibody (1:200, Invitrogen, #MA5-31332), mouse anti-α-SMA primary antibody (1: 200, Abcam, #ab7817), and rabbit anti-β-tubulin primary antibody (1: 200, Abcam, #ab15568). In addition, the hydrogels were incubated for 48 h with rabbit anti-PDGFR-β primary antibody (1:200, Abcam, #ab32570). The hydrogels were then labeled with the following secondary antibodies: goat anti-mouse Alexa Fluor 488 (1:200, Thermo Fisher, #A11029), and donkey anti-rabbit Alexa Fluor 549 (1:200, Thermo Fisher, #21207). DAPI (1 µg/ml, Sigma, #9542) was used to stain the nuclei. To observe the fluorescence signals from the cells, laser scanning confocal microscopy (LSCM, Olympus FV3000) and Olympus FluoView FV31S-SW software were applied. Images 5.0 μm thick in a z-stack were analyzed. All samples were imaged using identical exposure times and laser power settings. Qualification imaging analysis was performed using IMARIS 7.6 software.

## Results

### Fabrication of elastin-variable hydrogels with characterization by MNLO imaging

**Fig. 1:**
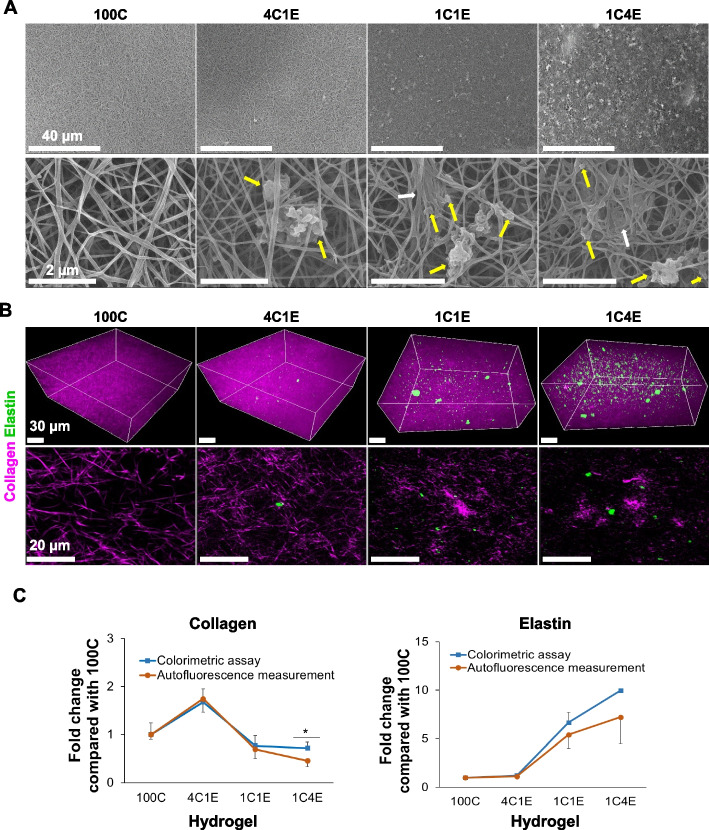
Morphology of elastin-variable hydrogels without cells at 0 h. **A** Scanning electron microscope (SEM) imaging of hydrogels without cells. The yellow arrows indicate insoluble elastin, and the white arrows point to clumps of collagen. **B** Multimodal nonlinear optical (MNLO) imaging of hydrogels without cells. The purple second harmonic generation (SHG) signal represents collagen, and the green two-photon excitation fluorescence (TPEF) signal represents elastin. **C** Levels of collagen and elastin in hydrogels at 0 h, as measured by colorimetric assays and autofluorescence intensity analysis. Data are represented as mean ± standard deviation (SD) of triplicate analyses. * *p* < 0.05

To develop 3D cellular environments with an elastin gradient, four types of hydrogels combining elastin and collagen were fabricated by controlling the pH and processing temperature without a chemical crosslinker. Since native elastin is difficult to be integrated into cellular environments due to the large protein size and insolubility, we optimized the procedure for suspending elastin in 0.1 M acetic acid. Figure 1A shows SEM and MNLO images of the four elastin-variable hydrogels (100C, 4C1E, 1C1E, 1C4E). As shown in Fig. 1A, collagen was imaged in a fibrillary structure, while elastin showed some clumps entangled with collagen fibers corresponding to the elastin concentration in 4C1E, 1C1E, and 1C4E. Based on the inherent properties of collagen and elastin, we performed label-free 3D nonlinear optical imaging (second harmonic generation and multi-photon excitation fluorescence). In Fig. 1B, the intensities of elastin (green) and collagen (magenta) in the hydrogels are shown according to the elastin/collagen ratios. In order to confirm the amount of collagen and elastin measured using MNLO imaging, the result was compared to a conventional colorimetric measurement method, as shown in Fig. 1C. According to the similarity of the results, MNLO imaging was used for collagen and elastin measurement in this study.

### Biofunctional properties of fibroblasts in elastin-variable hydrogels

**Fig. 2:**
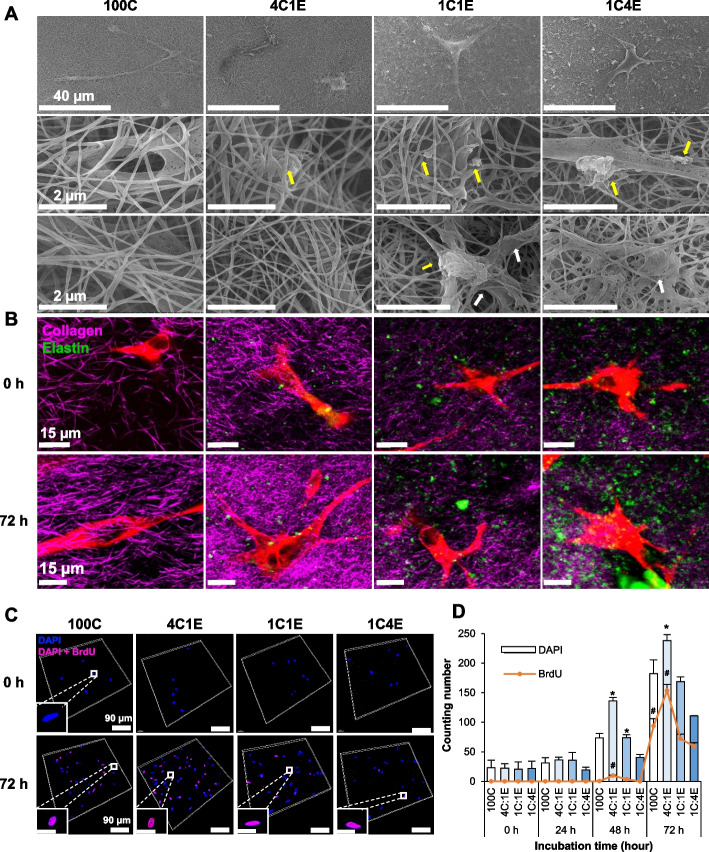
Morphology of cell-laden elastin-variable hydrogels. **A** SEM imaging of hydrogels with cells at 72 h. The yellow arrows indicate insoluble elastin, and the white arrows point to clumps of collagen. **B** MNLO imaging of hydrogels with cells at 0 h and 72 h. The purple SHG signal represents collagen, and the green TPEF signal represents elastin and autofluorescent molecules in cells. **C** Human dermal fibroblast (HDF) proliferation in hydrogels illustrated through z-stack confocal images of the BrdU assay, showing the DAPI signal (blue), BrdU signal (red), and a combination of DAPI and BrdU signals (purple). **D** Cell counting based on the DAPI signal (total cell number) and BrdU signal (proliferating cells). Data are represented as mean ± SD from triplicate analyses. *, # *p* < 0.05

To investigate the effects of elastin on fibroblasts in a 3D environment, we fabricated elastin-variable hydrogels laden with fibroblasts. For this, human dermal fibroblasts were incorporated at an optimal density (1 × 10^5^ cells/ml) before the gelation process. Fabricated hydrogels with and without HDFs were shown in Supplementary Information Fig. S1. The proliferation of fibroblasts in the fabricated hydrogels was studied in a prolonged culture condition of 72 h. Figure 2A shows SEM images of the fibroblast-laden elastin-variable hydrogels. HDFs were well integrated in collagen fibers, and some elastin clumps surrounding cells were observed in 4C1E, 1C1E, and 1C4E. The length of collagen fibers were measured showing decreased length in 1C4E (Fig. S2). Interestingly, the phenotype of the fibroblasts changed inside the elastin-variable hydrogels with changes in the collagen and elastin ratio. Figure 2B shows MNLO images of β-tubulin-stained cells from the elastin-variable hydrogels. Compared to the spindle morphology of fibroblasts in 100C, the fibroblasts in the elastin-containing hydrogels showed a stellate-shaped morphology, especially in 1C4E. In terms of cell count, the proliferated cells were significantly increased in 4C1E compared to the others, as shown in the BrdU assay in Fig. 2C and D. It is noteworthy that this hydrogel provides a cell-friendly environment and that fibroblasts differentially proliferate depending on the level of elastin.

### Proteomic analysis of HDFs in elastin-variable hydrogels

**Fig. 3:**
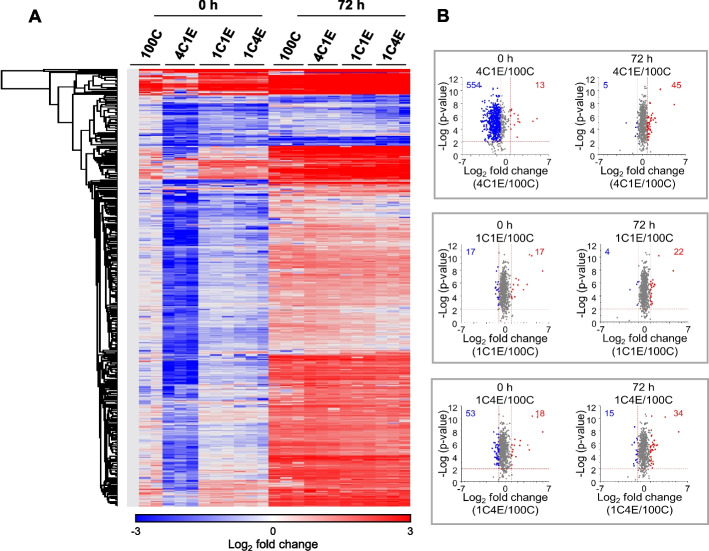
Label-free quantification-based proteomic analyses of HDFs in four elastin-variable hydrogels at early and late stages by nLC-ESI–MS/MS. **A** Heat map of the differentially expressed proteins with at least a ± 1.5-fold change. **B** Volcano plots of HDFs in elastin-variable hydrogels compared with control (100C). The results show the proteins log2 fold change plotted against the –log10 p-value. Blue and red colors in the heat map and volcano plots represent downregulated and upregulated proteins in the elastin-variable hydrogels compared to 100C, respectively. Red dotted lines indicate *p* = 0.01 and ± 1.5-fold change

We next explored the proteomic profiles in the fibroblast-laden elastin-variable hydrogels by performing nLC-ESI–MS/MS analysis. Four types of HDF-laden elastin-variable hydrogels after the 72 h culture were subjected to the proteomic analysis. To prevent the collagen and elastin proteins making up the hydrogels from interfering with the signal when detecting intracellular proteins, collagen and elastin were dissociated upon cell harvest. A total of 719 proteins were quantified, of which 558 proteins were over 1.5-fold changed compared to control. We visualized the upregulation or downregulation of the quantified proteins in a heat map to explore the changes in response to cell culture time and elastin gradient (Fig. 3A). Comparing the hydrogels at 0 h, the heat map shows that the overall proteins in the HDFs of 4C1E were significantly downregulated. Whereas after 72 h, the downregulated proteins tended to be upregulated regardless of the elastin concentration. A volcano plot of the quantified proteins allows us to see which proteins have both a large fold change (x-axis) and high statistical significance (-log10 of p-value, y-axis). As shown in Fig. 3B, 554 downregulated proteins of the 4C1E HDFs were identified, implying that the expression of proteins was inhibited at 0 h compared to other hydrogels. However, in all hydrogels at 0 h, the number of upregulated proteins of the HDFs was similarly small. After 72 h, the number of upregulated proteins slightly increased overall. Based on the volcano plots in Fig. 3B, at the low level of elastin (4C1E), significantly different proteomic changes occurred. Therefore, we focused on the effect of the low level of elastin in 4C1E compared to the 100C environment hereafter.

### Early-stage proteomic changes in the 4C1E hydrogel: calcium signaling

**Fig. 4:**
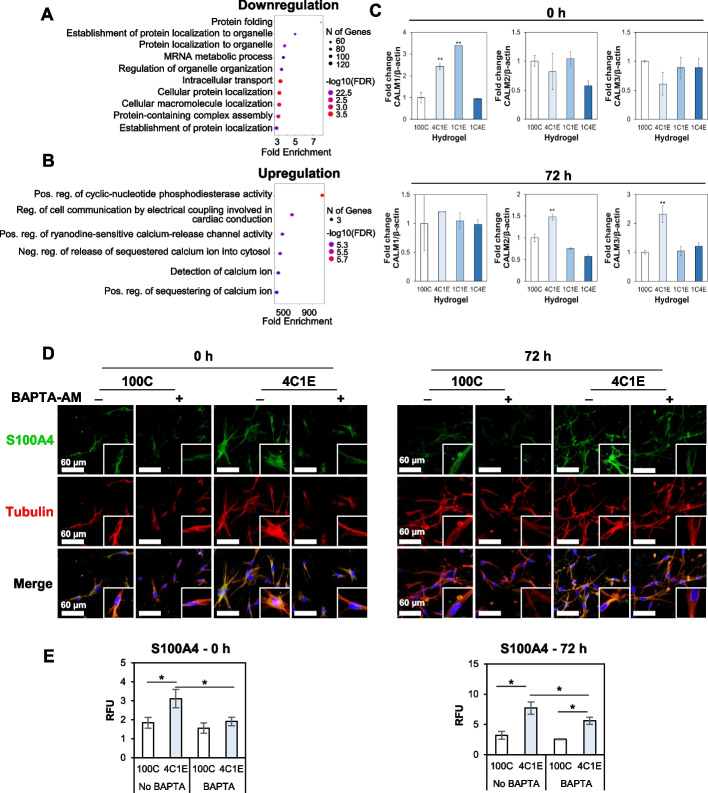
Early stage of proteomic changes. Bubble plots represent the biological processes of **A** downregulated and **B** upregulated proteins in the 4C1E hydrogel compared with 100C. The vertical axis shows the significantly enriched biological processes, and the horizontal axis represents the fold enrichment corresponding to the biological processes. Fold enrichment indicates the percentage of differentially expressed proteins divided by all proteins within a certain gene ontology (GO) term. **C** Expression of *CALM1,2,3* in HDFs within hydrogels at 0 h and 72 h, quantified via qRT-PCR. **D** Changes in Ca2 + related protein S100A4 (depicted in green) in HDFs (with tubulin depicted in red) with or without BAPTA-AM (100 μM) treatment in 4C1E, as compared to those in 100C, observed through confocal imaging. **E** Analytical data derived from the images in **D**. Data are represented as mean ± SD of triplicate analyses. * *p* < 0.05

Given that the proteomic expression in 100C and 4C1E showed differences between early and late stages, we performed proteomic analysis by nLC-ESI–MS/MS at both stages. First, proteomic results showed that the downregulated proteins of HDFs at 0 h in 4C1E were involved in protein folding, protein localization to organelles, and mRNA metabolic process (Fig. 4A). On the other hand, the upregulated proteins of HDFs were related to calcium ion activity such as calcium-release channel activity and calcium ion sequestering (Fig. 4B). Interestingly, in all biological processes of the upregulated proteins, three calmodulin (CaM) proteins were observed. CaM has a fully preserved amino acid sequence across all vertebrates and is structurally and functionally encoded by three genes (*CALM1*, *CALM2*, and *CALM3*) [27, 28]. For this reason, the three CaM proteins quantified by nLC-ESI–MS/MS had the same protein sequence and were thus difficult to distinguish initially. To determine which CaM proteins were activated in the HDFs in response to the four elastin concentrations, we performed qRT-PCR as a gene-level analysis (Fig. 4C). In the qRT-PCR data of the three *CALM* genes, the expression of *CALM1* was significantly increased in 4C1E and 1C1E at 0 h, after which it did not much change at 72 h (top of Fig. 4C). In contrast, *CALM2* and *CALM3* showed no significant change at 0 h, but it was confirmed that these genes in 4C1E increased at 72 h (bottom of Fig. 4C). These results suggest that CaM is produced by the *CALM1* gene in the early-stage HDFs with an altered dermal composition, and that they are activated in the biological pathways associated with calcium ions.

Since calcium signaling is presumably associated with the differentiation of fibroblasts in the elastin-variable hydrogel environments, we assessed the expression of S100A4 (also called fibroblast-specific protein 1, FSP1), which is a marker of differentiated fibroblasts as well as a calcium-binding protein. In Fig. 4D and 4E, fibroblasts in 4C1E showed increased S100A4 expression compared to cells in 100C. After treatment with BAPTA-AM, a calcium chelator, S100A4 was significantly decreased in 4C1E compared to 100C and non-treated cells. These results support the hypothesis of calcium ion participation in fibroblast differentiation under the presence of a low level of elastin at an early stage.

### Late-stage proteomic changes in the 4C1E hydrogel: ECM increase

**Fig. 5:**
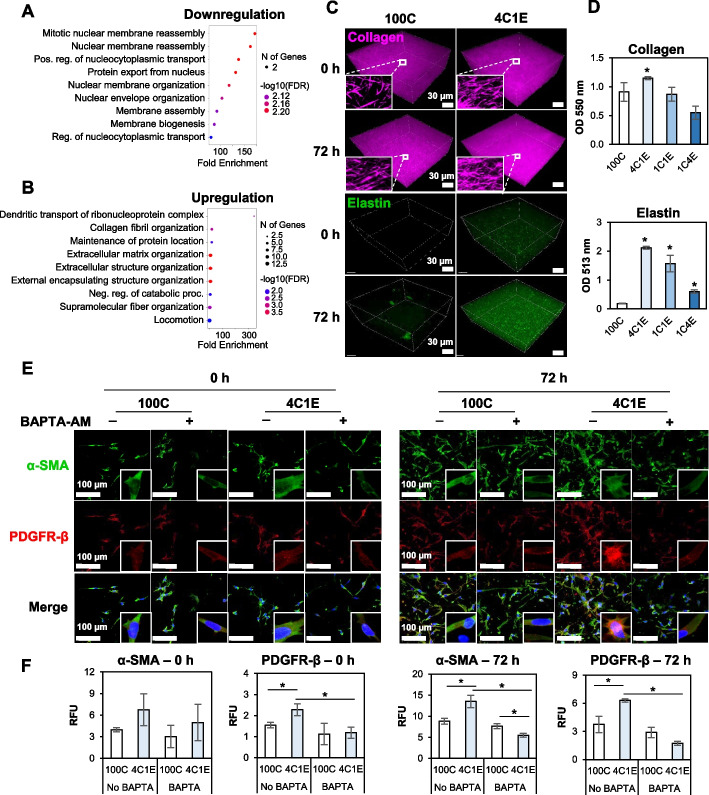
Late stage of proteomic changes. Bubble plots represent the biological processes of **A** downregulated and **B** upregulated proteins in 4C1E compared with 100C. The vertical axis shows the significantly enriched biological processes, and the horizontal axis represents the fold enrichment corresponding to the biological processes. Fold enrichment indicates the percentage of differentially expressed proteins divided by all proteins within a certain GO term. **C** MNLO imaging of hydrogels with cells at 0 h and 72 h. The purple SHG signal represents collagen, and the green TPEF signal represents elastin and autofluorescent molecules in cells. **D** Colorimetric measurement of generated collagen and elastin in hydrogels after 72 h. **E** Expression of α-SMA (green) and PDFGR-β (red) proteins in hydrogels with or without BAPTA-AM (100 µM) treatment at 0 h and 72 h. DAPI (blue) indicates the nuclei. **F** Analysis data derived from the images in **E**. Data are represented as mean ± SD of triplicate analyses. * *p* < 0.05

In the late stage of proteomic changes after 72 h incubation in 3D hydrogels, downregulated proteins were involved in mitotic nuclear membrane reassembly, positive regulation of nucleocytoplasmic transport, and protein export from the nucleus, as presented in the nLC-ESI–MS/MS results (Fig. 5A). On the other hand, the biological processes of the upregulated proteins were associated with collagen fibril organization, ECM organization, and supramolecular fiber organization (Fig. 5B). To validate these results, the generated amounts of collagen and elastin were quantified by measuring the MNLO intensities of the 3D hydrogels before and after the 72 h culture (Fig. 5C). In 4C1E, collagen and elastin were significantly produced, a result further confirmed by colorimetric analysis (Fig. 5D). Then PDGF-β, a CAF marker, and α-SMA, a myofibroblast marker, were further validated in the low elastin hydrogel (4C1E) by immunofluorescence staining (Fig. 5E and F). In the 4C1E hydrogel, PDGF-β and α-SMA were highly expressed in both early (0 h) and late (72 h) culture. Following the treatment of BAPTA-AM, the expression levels of PDGF-β and α-SMA were decreased, implying a relation to calcium signaling.

### Fibrotic disease-related proteins in the low elastin hydrogel

**Fig. 6:**
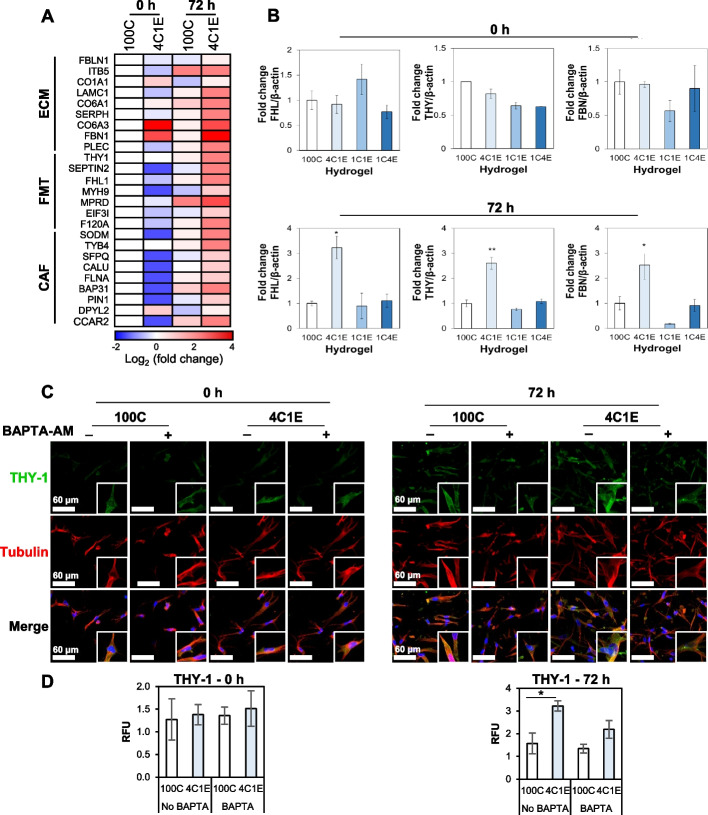
HDF-driven fibrotic disease-related proteins in the low elastin hydrogel. **A** Protein expressions of ECM-, FMT-, and CAF-related proteins in early and late stages. Blue and red squares represent downregulated and upregulated proteins in elastin-variable hydrogels compared to 100C in the early stage, respectively. **B** qRT-PCR analysis of FHL1, THY-1, and FBN1 gene expression levels in the hydrogels at 0 h and 72 h. **C** Expression of THY-1 protein (green) in HDFs (red) within hydrogels with or without BAPTA-AM (100 µM) treatment at 0 h and 72 h. **D** Analytical data derived from the images in C. Data are represented as mean ± SD of triplicate analyses. * *p* < 0.05

Since an increase in both ECM and S100A4 expression level is considered as a hallmark of myofibroblast differentiation, we investigated the proteins related to fibrotic diseases by proteomic profiling. The heat map displayed in Fig. 6A shows three categories of changed proteins, namely those related to the ECM, FMT, and cancer-associated fibroblasts or CAFs. First, there were a total of nine proteins related to the ECM, among which collagen alpha-1(I) chain (CO1A1) [29–31], collagen alpha-1(VI) chain (CO6A1) [32, 33], collagen alpha-3(VI) chain (CO6A3) [34, 35], and fibrillin-1 (FBN1) [36–39] were all shown to be upregulated in 4C1E compared to the control group regardless of incubation time. In particular, CO6A3 and FBN1 showed 4.2- and 12.9-fold higher levels of protein expression than the control, respectively. Second, a total of seven proteins were associated with the FMT. With the exception of Thy-1 membrane glycoprotein (THY-1) [40–42], these proteins were downregulated in the HDFs cultured in 4C1E at 0 h, whereas they were upregulated after 72 h. Finally, a total of nine proteins were related to CAFs. It can be noted here that most of the above-mentioned proteins are linked to tumor and systemic fibrosis. Characteristically, CO6A4 and FBN1 were initially upregulated as ECM-associated proteins, and this trend was sustained up to 72 h. By 72 h, all ECM, FMT, and CAF-related factors were upregulated across the board. The 4C1E environment not only encourages HDFs to produce an abundant ECM but can also be considered a potentially transformative environment for myofibroblasts or cancer.

Among the changed proteins, FBN1, THY-1, and four and a half LIM domains 1 (FHL1) were validated by qRT-PCR. Interestingly, at a low level of elastin (4C1E), the related genes were significantly increased at 72 h of culture (Fig. 6B). The expression of *THY1* was further validated by immunofluorescence staining. The level of *THY1* in the 72 h culture was significantly increased in the cells in the 3D hydrogel (Fig. 6C and D).

## Discussion

The development of a 3D cell-growing system close to native ECM has been a long-standing issue since the ECM microenvironment has a reciprocal relationship with cells to control physiological and pathological phenomena. In this study, we developed an elastin-gradient 3D hydrogel system suitable for long-lasting cell culture and demonstrated the effect of elastin on fibroblast differentiation, followed by a proteomic profiling analysis for a study of the molecular mechanism.

Elastin is one of the constituents of the ECM and serves as a regulatory protein of cell behavior. However, due to its insolubility and large molecular mass, the fabrication of elastin-containing hydrogels is relatively difficult compared to other ECM components. Many studies have utilized soluble elastin-derived peptides or tropoelastin fibers, but these might have some discrepancy in the cellular responses as elastin fibers in native tissue are insoluble [21]. Our elastin-gradient hydrogel system allowed us to integrate insoluble elastin by optimization of pH and temperature. Among the four different gradients (100C, 4C1E, 1C1E, and 1C4E), interestingly, the hydrogel with the low level of elastin (4C1E) showed the highest proliferation rate compared to the others in the 72 h culture (Fig. 2C and D). On the other hand, in the hydrogels with higher levels of elastin (1C1E, 1C4E), cellular proliferation was significantly decreased (Fig. 2C and D), and the generation of collagen and elastin was simultaneously decreased (Fig. 5D), implying abnormal cellular function. One explanation might be that the addition of elastin extensively densified the collagen, resulting in a shortened length of collagen fibrils and leading to a decrease in the available space to grow cells that hindered fibroblast proliferation [13]. The measurement of collagen fibril length is consistent with this explanation (Supplementary Information Fig. S2). In addition, in Fig. 1A and B, TEM and MNLO images of the elastin-variable hydrogels without cells showed collagen aggregations, or molecular crowding, in 1C4E (white arrows). By the addition of elastin, collagen fibers were entangled to form thick bundles with a maintained thickness of each single collagen fiber. This could be attributed to an increased collagen network density [13]. Although elastin can improve cellular attachment and proliferation in elastin-binding domains compared to collagen only [43], the increase in collagen network density has a much greater impact on the limited space for cellular growth in a 3D environment, making it difficult for cells to grow in 1C4E. Therefore, the subsequent analyses on the effect of elastin on fibroblast differentiation were conducted focusing on the lowest level of elastin, 4C1E, which provided the best condition for cell growth.

Using the 3D elastin-gradient hydrogel system, the elastin effect on fibroblast-to-myofibroblast differentiation was evidenced by the following: 1) a morphological change, 2) an increase in the production of ECM, and 3) an increase in myofibroblast markers validated by protein profiling analysis, qRT-PCR, and immunocytochemistry. First, the shape of embedded HDFs in the hydrogel changed to a stellate morphology, indicating the characteristic feature of myofibroblasts (Fig. 2B). This was demonstrated by MNLO imaging, which was nondestructively performed in 3D. Then, newly synthesized elastin and collagen inside the hydrogels were increased, which is one of the consequences of myofibroblast differentiation (Fig. 5C and D). This was also validated through MNLO imaging–based quantification with confirmation by conventional colorimetric methods.

Differentiation to myofibroblasts proceeded in early and late phases in the 3D elastin-gradient hydrogel system. We performed proteomic analysis on the HDF-laden elastin-variable hydrogels at the early stage (right after the 2 h gelation period before incubation) and the late stage (after 72 h incubation). At the early stage, in the 4C1E hydrogel, the downregulated proteins were associated with regulation of organelle organization. These responses can contribute to the pathogenesis of human diseases [44, 45]. On the other hand, calcium ion–related proteins were upregulated, including CaM produced by the *CALM1* gene. In calcium signaling, CaM plays crucial functions in all eukaryotic cells and is involved in the regulation of numerous calcium-dependent pathways in growth, proliferation, and migration [46]. This result with increased proliferation in 4C1E agrees with previous studies that calcium may promote fibroblast proliferation [47], and abnormal expression of CaM in cells may be a sign of fibrosis [48]. Moreover, S100A4, a marker for differentiated fibroblasts as well as a calcium-binding protein, was decreased by the treatment of a calcium chelator, BAPTA-AM, supporting the calcium-mediated responses involved in fibroblast differentiation (Fig. 4D and E). These calcium-related responses were revealed as an acute event right after gelation. It should be noted that these initial calcium-mediated responses may influence the subsequent FMT phenomena such as integrin- and THY-1-mediated differentiation.

Meanwhile, at the late stage after 72 h cultivation, further evidence was found that low-level elastin promoted fibroblast differentiation. After culturing HDFs for 72 h in 4C1E, the downregulated proteins were involved in nucleus-related roles. A previous report has shown that the subcellular organization of fibroblasts depends on the surrounding environment, and that alteration leads to changes in subcellular nuclear polarity and location [4]. Thus, the downregulated pathways in both early and late stages may have a direct impact on pathological conditions or the FMT. On the other hand, the upregulated proteins in the late stage were related to alterations of the ECM. Since TGF-β is well known to induce FMT, we confirmed that our results of ECM increase were consistent with FMT caused by induction of TGF-β (Supplementary Information Fig. S3) [36]. This result suggests that the hydrogel containing low-level elastin stimulated the production of ECM in the surrounding environment via fibroblasts.

Along with an increase in generated ECM as one of the phenomena by differentiated myofibroblasts, moreover, the increased expression of proteins associated with the FMT including THY-1, FHL1, and FBN1 was observed by mass spectrometry analysis and qRT-PCR (Fig. 6A and B) [40, 49]. Although no significant changes in THY-1, FHL1, or FBN1 were observed in the early analysis stage, it is noteworthy that these proteins were expressed after several passages of cultivation in the insoluble elastin-containing 3D hydrogel. Furthermore, we performed calcium chelator treatment on the expression of THY-1 and found that calcium ions influenced the THY-1 protein after 72 h of incubation (Fig. 6C and D), which is consistent with the real-time PCR results. There was a significant decrease in both α-SMA and PDGFR-β expression after 72 h in the BAPTA-AM pretreated fibroblasts compared to non-treated fibroblasts (Fig. 5E and F). These results can be interpreted that the FMT can be effectively controlled by early calcium restriction, which may suggest the potential for establishing therapeutic strategies.

Elastin is known to functionally promote cell proliferation and adhesion and to structurally incorporate into other ECM composites containing collagen. In our 3D elastin-gradient hydrogel system, the FMT was mainly demonstrated in the 20% elastin environment (4C1E). However, increases in incorporated elastin did not correlate with an increase in cell proliferation or the FMT, especially in 1C4E. This is due to an over-densified collagen network by the incorporated elastin, leading to insufficient space for cellular growth, as shown in Fig. 1. Therefore, high concentrations of elastin did not increase cell proliferation or the FMT.

**Fig. 7:**
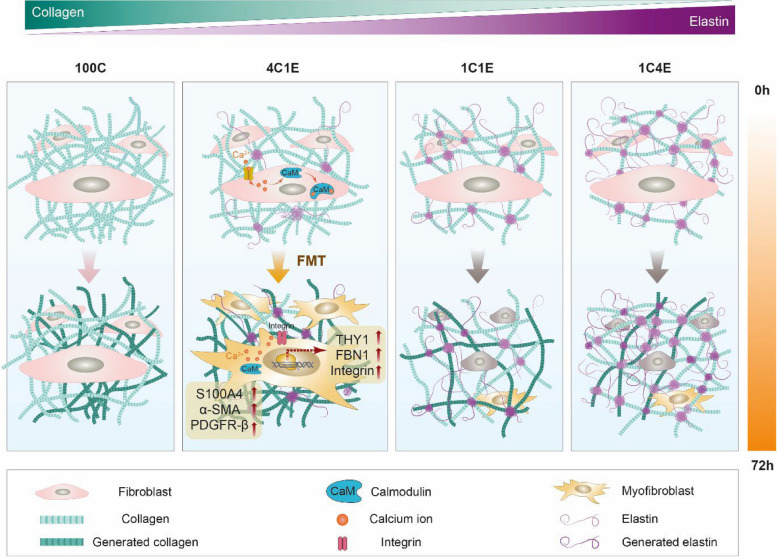
Model of the FMT on elastin-variable 3D hydrogel environments. Among the four types of elastin-variable hydrogels (100C, 4C1E, 1C1E, and 1C4E), 20% elastin in 4C1E induces the FMT via calcium signaling. The FMT is demonstrated by an increase in collagen synthesis and increased expression of biomarkers (S100A4, PDFGR-β, THY-1, FHL1, FBN1, and α-SMA).

In 4C1E, the FMT was induced via mechanical sensing molecules including integrin, FBN, and THY-1. Integrin, a cellular surface molecule that interacts with the ECM, provides a dynamic link between the ECM and intracellular signaling. In our proteomic analysis and immunofluorescence staining results (Supplementary Information Figs. S4 and S5), integrin was highly expressed in 4C1E, implying a changed level of cellular binding sites of collagen according to a different elastin–collagen composite configuration. THY-1, a well-known marker of fibroblasts to implicate fibrosis, has been studied little in skin fibrosis compared to many studies including neuron- and T-cell-related immunities. Recently, it was demonstrated that THY-1-positive myofibroblasts played a pathogenic role as a biomarker of scleroderma [40]. In our study, THY-1 was highly expressed in 4C1E compared to the other hydrogels, consistently found from gene expression to immunostaining. Since THY-1 can be activated by integrin-mediated signaling to collagen, this result aligns with our measurement showing that the highest amount of collagen was in 4C1E, not 100C (Fig. 5C and D). Since mechano-sensing molecules such as integrin, THY-1, and FBN-1 were increased, the modulus of the elastin-gradient hydrogel system was measured (Supplementary Information Fig. S6). The modulus of 4C1E was highly increased, but other elastin-containing hydrogels (1C1E and 1C4E) showed only slightly decreases compared to 100C. This is due to the property of insoluble elastin and agrees with previous reports [21]. Since the FMT occurred in 4C1E (20% elastin) at which the modulus is high, the modulus could partially affect the FMT. According to these results, even though an increase in elastin concentration can increase cellular responses including cell proliferation and differentiation, physical changes in the collagen network by increasing elastin dominantly affect the FMT. It is noteworthy that 20% elastin in 4C1E optimally densified the collagen network, resulting in increased THY-1 and integrin expression in fibroblasts, leading to the FMT. Our data showed that 20% elastin could induce the pathogenic differentiation of dermal fibroblasts by remodeling the ECM microenvironment (Fig. 7).

## Conclusion

In this study, we developed a 3D hydrogel system containing insoluble elastin in different gradients for long-term cell culture and demonstrated that 20% elastin induces the FMT, and thus can be considered as a pathological condition by remodeling the cellular microenvironment. Using nonlinear optics imaging, the extent of ECM change was quantitatively analyzed in 3D as well as in a noninvasive and label-free manner. Furthermore, mass spectrometry analysis revealed associated protein expressions throughout the long-lasting cell culture. Owing to the highly biocompatible and cell-friendly conditions of our elastin-gradient system, the effects of 20% elastin could be explicitly investigated in early and late stages. We demonstrated that calcium signaling in the early stage influenced the FMT in the late stage. For the 20% elastin hydrogel, results showed that various biomarkers, including myofibroblast cell markers (α-SMA, THY-1) and cancer-associated markers (PDGF-β, S100A4), were involved in the FMT. Overall, our elastin-gradient system can provide a versatile ECM platform for other cells that need a 3D environment containing insoluble elastin.

### Supplementary Information

**Additional file 1.**

## Data Availability

Data sharing is not applicable to this article.

## Abbreviations

ECM: Extracellular matrix
3D: Three-dimensional
2D: Two-dimensional
CAF: Cancer-associated fibroblast
FMT: Fibroblast-to-myofibroblast transition
α-SMA: α -Smooth muscle actin
100C: 100% Collagen
4C1E: 80% Collagen: 20% elastin
1C1E: 50% Collagen: 50% elastin
1C4E: 20% Collagen: 80% elastin
CS: Collagen stock solution
ES: Elastin stock solution
TGF-β: Transforming growth factor-β
BrdU: 5-Bromo-2-deoxyuridine
SHG: Second harmonic generation
HDF: Human dermal fibroblasts
MNLO: Multimodal nonlinear optical
SEM: Scanning electron microscope
CaM: Calmodulin
nLC-ESI–MS/MS: Nanoflow liquid chromatography–electrospray ionization–tandem mass spectrometry
