# Supplementary material for: Time-sequential fibroblast-to-myofibroblast transition in elastin-variable 3D hydrogel environments by collagen networks
Source: Biomater Res. 2023 Oct 17;27:103. doi: 10.1186/s40824-023-00439-x (PMC10583321; doi:10.1186/s40824-023-00439-x)
Supplement: Supplementary file 1 — Additional file 1. [file 40824_2023_439_MOESM1_ESM.docx]

**Supplementary File**

Time-sequential fibroblast-to-myofibroblast transition in elastin-variable 3D hydrogel environments by collagen networks

*Nhuan T. Do, ^a,b^*^†^ *Sun Young Lee,^a^*^†^ *Yoon Seo Lee, ^a^*^†^ *ChaeHo Shin^c d^, Daeho Kim^e^,*

*Tae Geol Lee,^a,d^ Jin Gyeong Son,^a*^ and Se-Hwa Kim^a,b*^*

**
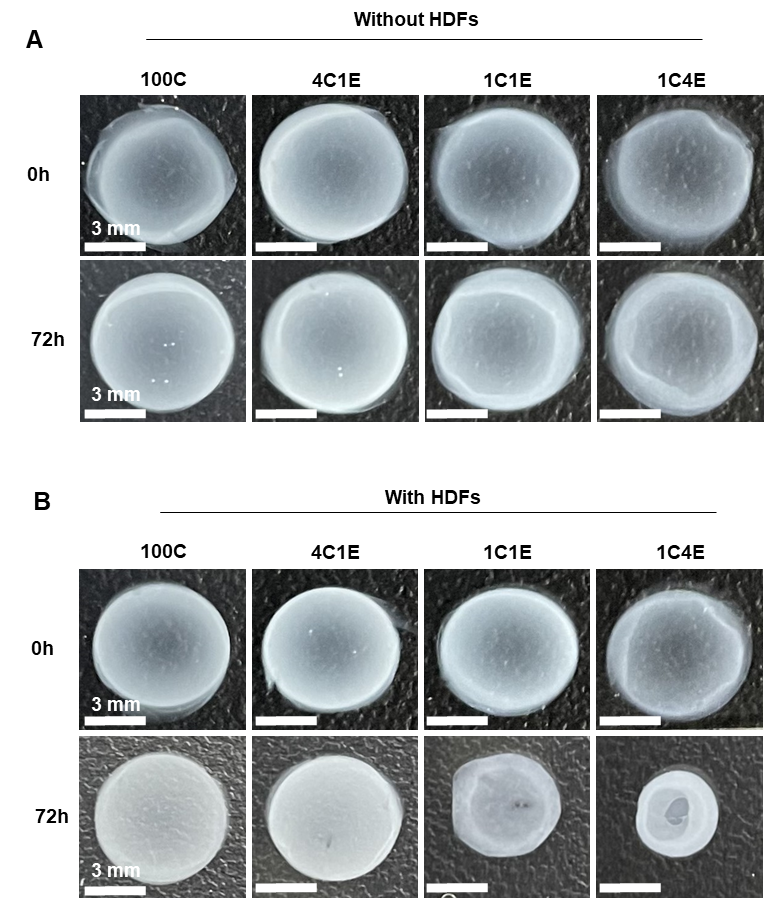
**

**Supplementary Fig. S1.** Optical images showing the appearance of elastin-variable hydrogels (A) without human dermal fibroblasts (HDFs) and (B) with laden HDFs at 0 h and 72 h.


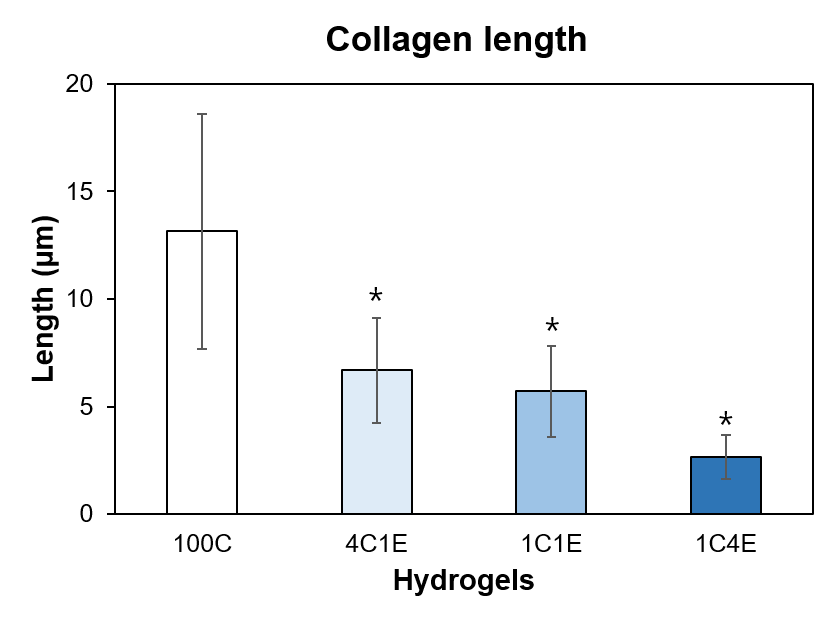


**Supplementary Fig. S2.** Collagen fiber lengths based on multimodal nonlinear optical (MNLO) images. Approximately 25 random fibers were selected for each measurement. One collagen fiber was judged as an independent line between two cross-links (or intersections) in the field of view (FOV). To measure the length of the collagen fibers, multiple 2D images were randomly chosen among z-stacked 3D images, and 25 fibers in one FOV were selected and the individual fiber lengths were measured. The data are represented as mean ± standard deviation (SD) of triplicate analyses. * *p* < 0.05


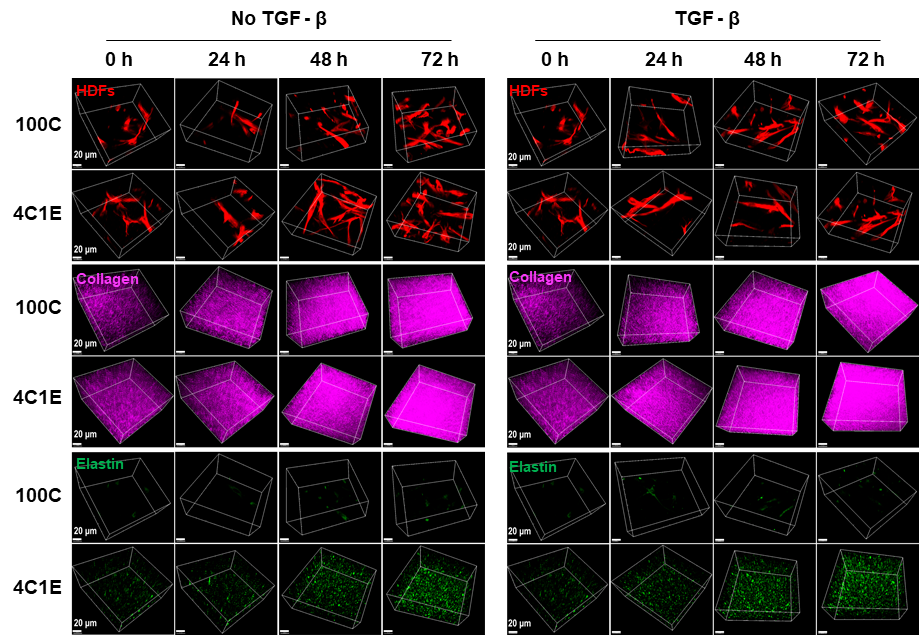


**Supplementary Fig. S3.** Effect of a low level of elastin on HDFs growing in 3D hydrogels using MNLO imaging methods from 0 h to 72 h comparing conditions (A) without TGF-β treatment and (B) with TGF-β treatment. The red tubulin represents growing HDFs, the purple second harmonic generation (SHG) signals represents collagen, and the green MNLO signals represent elastin and autofluorescent molecules in cells.


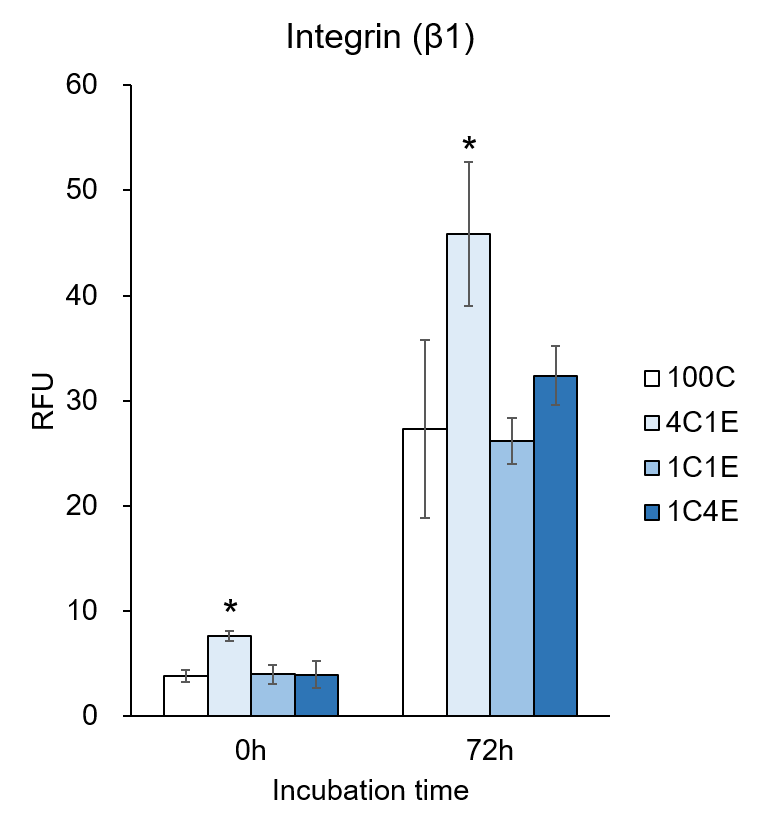

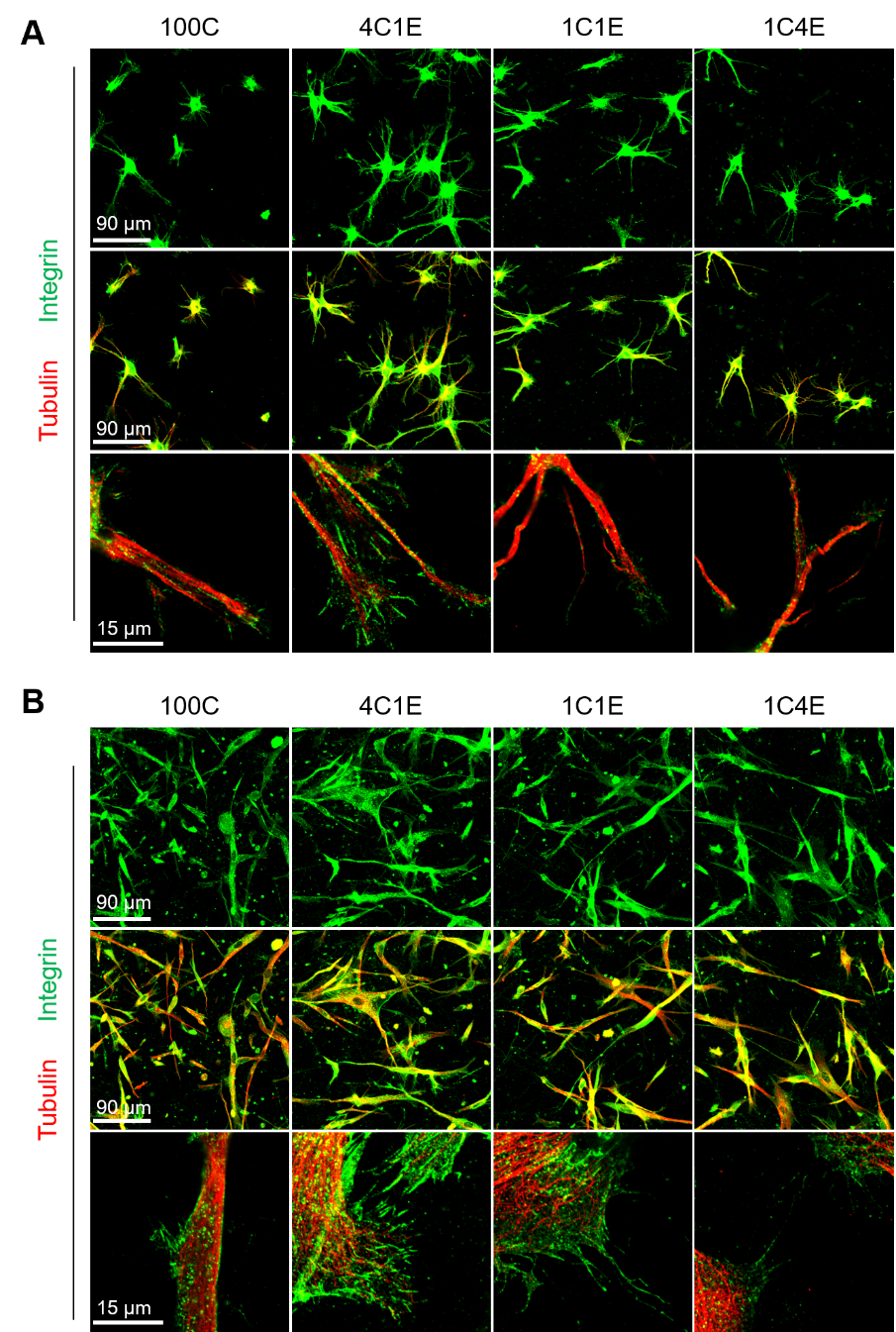


**C**

**Supplementary Fig. S4.** Expression of integrin in embedded HDFs in the elastin-gradient hydrogel system. (A, B) Signals from cell-laden elastin-variable hydrogels incubated at 0 h (A) and 72 h (B). Green indicates integrin and red indicates tubulin. (C) Quantification of expressed integrin levels based on the imaging results in A and B.

**Supplementary Fig. S5.** Expression of integrin-related proteins in the elastin-gradient hydrogel system using nLC-ESI-MS/MS based label-free quantification. The data are represented as mean ± standard deviation (SD) of triplicate analyses. **p* < 0.001


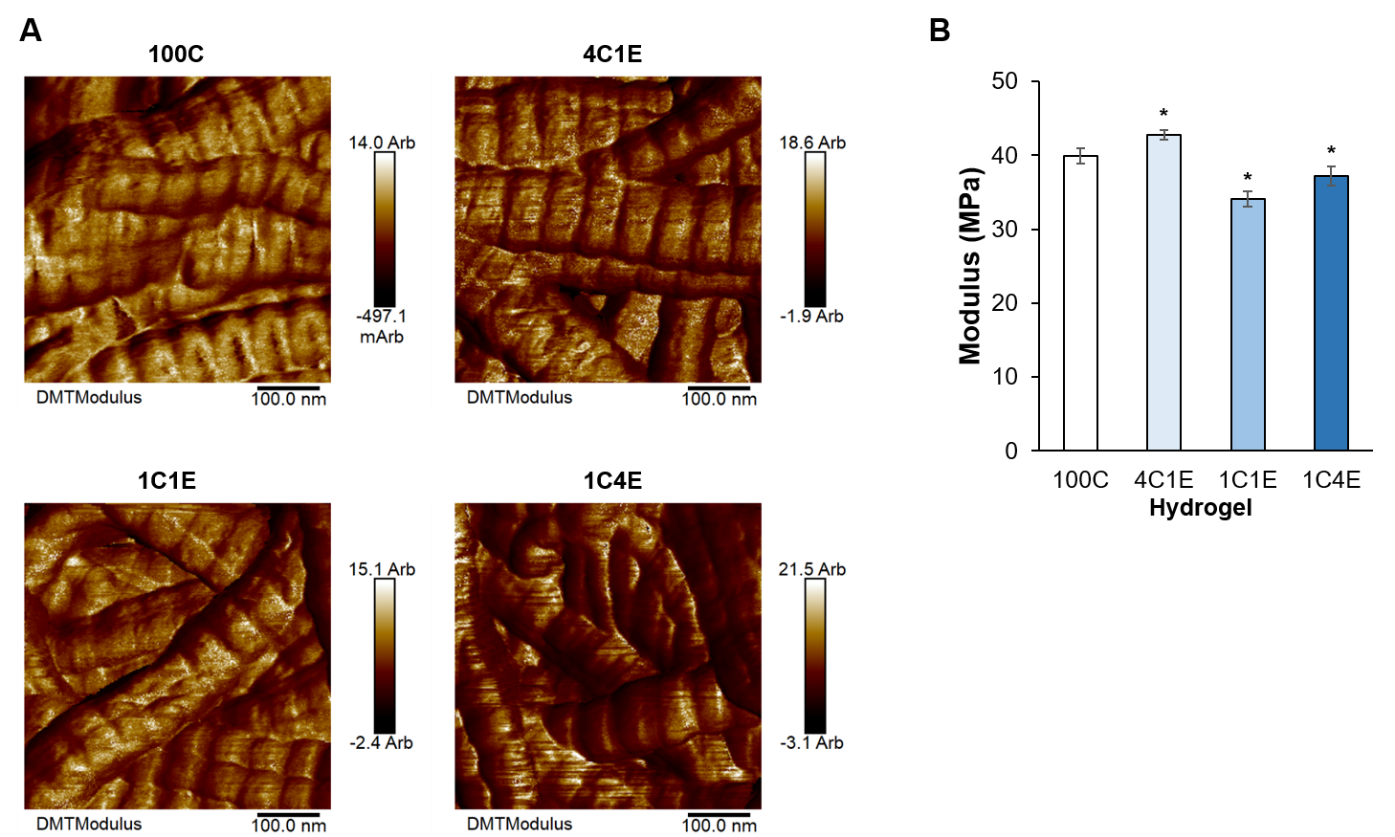


**Supplementary Fig. S6.** Modulus measurement by atomic force microscopy (AFM). (A) Modulus mapping and (B) histogram of the DMT modulus of the four elastin-variable hydrogels with laden HDFs at 0 h. Each elastin-variable hydrogel with laden HDFs at 0 h was dried at room temperature and used for measurement. The AFM (Dimension Icon, Santa Barbara, CA, USA) measurements of the mechanical properties of the samples were conducted in PeakForce QNM mode using a ScanAsyst-Air probe (Bruker, Santa Barbara, CA, USA) with a tip radius of 2 nm and 0.4 N/m spring constant. During AFM measurement, the peak force setpoint was set to 2 nN, and the total indentation depth was about 5 nm. The data was processed using a DMT model with a Poisson ratio of 0.3 to determine the Young’s moduli of the samples. The data are represented as mean ± standard deviation (SD) of quintuplicate analyses. **p* < 0.05
